# Histological correlates of postmortem ultra-high-resolution single-section MRI in cortical cerebral microinfarcts

**DOI:** 10.1186/s40478-020-00900-1

**Published:** 2020-03-13

**Authors:** Deniz Yilmazer-Hanke, Theresa Mayer, Hans-Peter Müller, Hermann Neugebauer, Alireza Abaei, Angelika Scheuerle, Joachim Weis, Karin M. E. Forsberg, Katharina Althaus, Julia Meier, Albert C. Ludolph, Kelly Del Tredici, Heiko Braak, Jan Kassubek, Volker Rasche

**Affiliations:** 1 Clinical Neuroanatomy, Department of Neurology, Institute for Biomedical Research, Ulm University, Helmholtzstr. 8/1, 89081 Ulm, Germany; 2 Neurology Clinic, Department of Neurology, School of Medicine, Ulm University, Ulm, Germany; 3 Core Facility Small Animal MRI, Medical Faculty, Ulm University, Ulm, Germany; 4 Department of Internal Medicine II, University Hospital Ulm, Ulm, Germany; 5 Department of Pathology, Section Neuropathology, University Hospital Ulm, Ulm, Germany; 6 Institute of Neuropathology, RWTH Aachen University Hospital, Aachen, Germany; 7 Department of Medical Biosciences & Pathology, Umeå Universitet, Umeå, Sweden

**Keywords:** Post-mortem magnetic resonance imaging, Histological matching, Microbleeds, String vessels, Cerebral microangiopathy, Small vessel disease

## Abstract

The identification of cerebral microinfarctions with magnetic resonance imaging (MRI) and histological methods remains challenging in aging and dementia. Here, we matched pathological changes in the microvasculature of cortical cerebral microinfarcts to MRI signals using single 100 μm-thick histological sections scanned with ultra-high-resolution 11.7 T MRI. Histologically, microinfarcts were located in superficial or deep cortical layers or transcortically, compatible with the pattern of layer-specific arteriolar blood supply of the cerebral cortex. Contrary to acute microinfarcts, at chronic stages the core region of microinfarcts showed pallor with extracellular accumulation of lipofuscin and depletion of neurons, a dense meshwork of collagen 4-positive microvessels with numerous string vessels, CD68-positive macrophages and glial fibrillary acidic protein (GFAP)-positive astrocytes. In MRI scans, cortical microinfarcts at chronic stages, called chronic cortical microinfarcts here, gave hypointense signals in T1-weighted and hyperintense signals in T2-weighted images when thinning of the tissue and cavitation and/or prominent iron accumulation were present. Iron accumulation in *chronic* microinfarcts, histologically verified with Prussian blue staining, also produced strong hypointense T2*-weighted signals. In summary, the microinfarct core was occupied by a dense microvascular meshwork with string vessels, which was invaded by macrophages and astroglia and contained various degrees of iron accumulation. While postmortem ultra-high-resolution single-section imaging improved MRI-histological matching and the structural characterization of chronic cortical cerebral microinfarcts, miniscule microinfarcts without thinning or iron accumulation could not be detected with certainty in the MRI scans. Moreover, string vessels at the infarct margin indicate disturbances in the microcirculation in and around microinfarcts, which might be exploitable in the diagnostics of cortical cerebral microinfarcts with MRI in vivo.

## Introduction

Cerebral microinfarcts are found in the brains of elderly, in cognitive impairment and dementia of vascular origin, and in patients with various neurodegenerative diseases such as Alzheimer’s or Parkinson’s disease [2, 12, 52]. Despite growing evidence for the contribution of cortical cerebral microinfarcts to the development of cognitive deficits and dementia, their identification in conventional magnetic resonance imaging (MRI) remains challenging [9, 21, 26, 29, 42]. Owing to their small size, cortical microinfarcts often remain below the detection limit of the image resolution in conventional MRI at 1.5 T and 3 T, although the detection rate of microinfarcts was improved in vivo with 7 T MRI [28, 45, 48]. Furthermore, the large size of the human brain makes the histological sampling and detection of cerebral microinfarcts difficult in routine paraffin sections [12, 48].

Postmortem magnetic resonance imaging (MRI) can help to overcome the obstacles encountered in the detection of structural changes in the brain with in vivo MRI, because it allows the use of higher field strengths and longer acquisition times with a better image resolution and enhanced signal-to-noise ratio [19, 36, 41]. Although the detection of cortical cerebral microinfarcts could be improved using postmortem 7 T MRI, artefacts still caused false positive results and some histologically verified microinfarcts remained undetectable in MRI scans even upon re-evaluation of the images [46, 49]. Currently it is unknown whether these cortical cerebral microinfarcts escaped detection and produced false negative results in MRI scans due to problems associated with technical limitations in image resolution at 7 T or MRI-histological matching [1, 37], or whether there is a subset of microinfarcts that produces a different MRI signal pattern.

The aim of this study, therefore, was to correlate structural changes in cortical cerebral microinfarcts at both MRI and histological level. For this purpose, we first performed a thorough analysis of morphological features of cortical cerebral microinfarcts by focusing on their shape and location, their iron load, and patterns of microvascular anomalities in thick brain sections. To overcome challenges that result from histological matching of microinfarcts to MRI scans [1], in a next step we developed a new ultra-high-resolution postmortem MRI technique by imaging single thick histological sections with proven microinfarcts with MRI at 11.7 T. Using this approach, we then compared MR signals produced by microinfarcts directly to morphological correlates of microinfarcts by staining the MR-imaged sections with various histological techniques. For a general histopathological characterization of microinfarcts, sections were stained with methods recently developed in our laboratory that visualize extracellular lipofuscin granules indicative of neuronal cell death, aldehyde fuchsine-positive macrophages, and alterations in the capillary network of microinfarcts [8]. MRI signals obtained in microinfarcts were further correlated with iron histochemistry and other histological alterations in MRI-scanned and adjacent sections. Microvascular changes and glial pathology were visualized using double-label immunohistochemistry for collagen together with markers of the vascular endothelium, astroglia or macrophages [22].

## Material and methods

### Subjects and neuropathological evaluation of the brain

Tissue from autopsy cases preserved at the Ulm University Tissue Bank underwent routine neuropathological examination. Thick brain sections used for diagnostics were screened for microinfarcts. Ten cases with cortical cerebral microinfarctions (5 females and 5 males, age range 69–89 years) were randomly selected for the study (for details: Table 1). Cases in the study cohort studied had Alzheimer’s disease (AD)-related argyrophilic neurofibrillary changes (NFT) at limbic stage 3 or less except case 8 (89 year old female with mixed dementia at NFT stage 4, MMSE 15/30, CDR 1.0) and varying degrees of extracellular Aβ deposition at cortical amyloid stages 0 (three cases) to A-C (seven cases) [6]. Staging of neuronal alpha-synuclein pathology [7] indicated Parkinson’s disease (PD) stage 0 in all cases except in case 10 (74 year old female at Parkinson’s stage 4). None of the cases were diagnosed with other tauopathies or alpha-synucleinopathies. This retrospective study was conducted in compliance with the university ethics committee guidelines as well as German federal and state law governing human tissue usage and in accordance with the Declaration of Helsinki. Informed written permission was obtained from all patients and/or their next of kin for autopsy.

**Table 1:** Demographic data and diagnoses of patients

Case	Age	Gender	Clinical Diagnosis	Cause of Death	Cortical Aβ Stage	CAA	NFT Staging (Gallyas/AT8)	PD Stage	Blocks used for screening	# MRI scanned sections/# MIs found	Location of Microinfarcts
1	84	F	Subacute cerebral MCA and PCA infarctions R	Pneumonia	0	0	2/3	0	Frontal, Hemipshere, Occipital ^b^	2/2	Several in areas around the hemorrhage
2	76	M	Heart failure	Cardiac arrest	C	0	0/1	0	Frontal, Hemipshere, Occipital	2/1	Frontoparietal transition (post. Frontolateral.)
3	77	M	Subcortical SVD	Pulmonary embolism	0	0	0/1	0	Frontal, Hemipshere, Occipital		Ant. frontal lobe (inf.frontal)
4	72	M	Cerebral white matter microangiopathy	Unknown	0	0	2/3	0	Frontal, Hemipshere, Occipital	2/1	Parietooccipital
5	70	F	Subcortical SVD, arterial hypertension	Intracerebral bleeding in frontoparietal lobe	C	0	0/1	0	Frontal, Hemipshere, Occipital ^c^		Middle parietal, post. Cingulate, sup. Temporal, occipital
6	83	M	Subcortical SVD, hydrocephalus	Aspiration pneumonia	B	0	2/3	0	Frontal, Hemipshere, Occipital		Frontal lobe (ant. Sup. frontal, middle frontal, inf. frontal)
7	69	M	Coronary artery sclerosis	Acute heart failure	B	0	1/3	0	Frontal, Hemipshere, Occipital	3/0	Frontal lobe (ant. Sup. frontal), occipital pole
8	89	F	Mixed dementia, arterial hypertension, subcortical SVD, TIA, Aneurysm of ACA, comp. CRF	Acute renal failure with bradycardiac arrhythmia	C, ^a^	Lpm	4/5	0	Frontal, Hemipshere, Occipital	1/2	Ant. frontal, lat. Inf. parietal
9	84	F	Arterial hypertension	Ischemic left MCA infarction	C	Lpm	3/4	0	Frontal, Hemipshere, Occipital		Ant. sup. Frontal
10	74	F	Parkinson’s disease	Pulmonary embolism	B	0	2/3	4	Frontal, Hemipshere, Occipital		Hippocampus

Alzheimer-related cortical Aβ deposits and neurofibrillary (NFT) changes were classified using the Braak staging (Acta Neuropathol 1991;82:239–59). Cortical and vascular Aβ deposits were visualized with the Campbell-Switzer silver stain and the anti-Aβ antibody 4G8. NFT staging was performed with the Gallyas silver stain and immunohistochemistry (IHC) with the anti-hyperphosphorylated tau antibody AT8. Parkinson staging was performed according to Braak (Neurobiol Aging 2003;24:197–211). *ACA* anterior cerebral artery, *comp. CRF* compensated chronic renal failure, *Lpm* leptomeningeal cerebral amyloid angiopathy (CAA), *MIs* Microinfarcts, *SVD*, small vessel disease, *TIA* transitory ischemic attack, ^a^Majority of isocortical Aβ deposits diffuse. "One section was imaged twice. ^b^Case with 13 blocks from the right and 16 blocks from left hemisphere; ^c^Case with 10 blocks from right hemisphere

### Histological processing and diagnostics

Brains were fixed in a 4% solution of formaldehyde and cut in approximately 1 cm thick coronal slices. Tissue slices containing frontal, mid-hemispheric, occipital and cerebellar blocks as well as blocks of the brainstem (rostral medulla, pontine-mesencephalic junction and midbrain) were embedded in polyethylene glycol (PEG 1000, Merck, Carl Roth Ltd., Karlsruhe, Germany). In addition, multiple coronal hemispheric blocks were available in 2 cases (Table 1). Several 100 μm thick consecutive sections were cut from each block with the aid of a sliding microtome (Jung, Heidelberg, Germany). Alzheimer-related neurofibrillary changes and extracellular deposits of Aβ peptide were visualized with advanced silver staining methods [6, 23] and immunohistochemistry with the mouse anti-phospho-tau (Ser202, Thr205) antibody (1.2000, clone AT8, Fisher Scientific GmbH, Schwerte, Germany) and mouse anti-β-amyloid (Aβ) 17–24 antibody (1:5000, clone 4G8, BioLegend, Koblenz, Germany). Alpha-synuclein pathology was detected with the anti-syn-1 antibody (1:2000, clone number 42; BD Biosciences, CA, USA).

For screening of microinfarcts, sections obtained from each coronal block were stained with the pigment Nissl stain (PN) using aldehyde fuchsine and Darrow red [4] in all cases. Additional sections stained with a modified hematoxylin eosin (H&E) procedure [22] were available in 8 cases (sections from all blocks in cases 1 & 5; 12 sections in the other 6 cases), and iron accumulation was visualized with Prussian blue staining [35] in 5 cases (16 sections). Free-floating sections adjacent to sections with proven microinfarcts were selected for MRI scanning and further histological analysis.

### Single section ultra-high resolution MR imaging

**Fig. 1:**
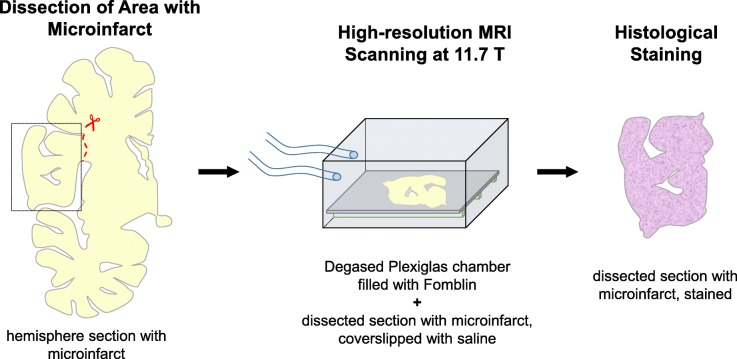
Procedure used to scan individual free-floating 100 μm thick sections with ultra-high-resolution MRI. The part of the section containing the microinfarct was dissected from the hemisphere section, coverslipped with saline, and imaged in a custom-made Plexiglas chamber in the MRI scanner. After completion of the scans, the section was processed for histological staining

Magnetic resonance (MR) images were taken at room temperature on an 11.7 T, horizontal bore (160 mm diameter) dedicated small animal system (BioSpec 117/16, Bruker Biospin, Ettlingen, Germany) equipped with BGA9 shielded gradients. The signal was recorded with a 72 mm quadrature volume transmit/receive resonator. Before imaging, the 100 μm-thick brain sections were stretched on glass object slides and coverslipped in a 0.9% NaCl saline solution by paying attention to minimize trapping of air bubbles (Fig. 1). Next, object slides with the sections were sealed tightly to prevent penetration of air or external solutions to the zone around the sections or to cavitated areas within the sections. The sealed object slides were placed in a custom-built Plexiglas chamber. The chamber was degased and filled with Fomblin (Sigma, St Louis, MO, USA). The samples were placed in the iso-center of the magnet. Multi-contrast three-dimensional (3D) data was obtained, including: T1-weighted (TR = 500, TE = 11.5), T2-weighted (TR = 3500, TE = 45) and proton density (PD, TR = 2000, TE = 8.5) single spin echo images with one signal average (NSA = 1) as well as gradient echo T2*-weighted images (TR = 24, TE = 12, flip angle =30°) with NSA = 16. Spatial resolution was as 100x110x200μm^3^. The single section ultra-high-resolution MRI technique was first established by scanning 3 sections without microinfarcts. In a next step, 10 sections with microinfarcts from 5 cases were imaged with the MRI scanner. In one case (case 4), two neighboring sections from the same microinfarct were imaged. In another case (case 1), one of the sections was imaged twice, confirming the reproducibility of MRI signals produced by the same microinfarct (for details: Table 1).

### Immunohistochemistry (ICH)

Sections imaged with MRI and adjacent sections (free-floating, 100 μm thick) were first treated with 10% methanol and 3% concentrated H_2_O_2_ in Tris-buffered saline (TBS) and then with bovine serum albumin (BSA). Antigen retrieval was performed using Tris-EDTA buffer at pH 9.0 or citrate buffer at pH 6.0 for 1/2 h at 100 °C or pretreatment with 1.3 μg/ml proteinase K for 10–15 min at 37 °C (Invitrogen, Darmstadt, Germany). The sections were incubated with primary antibodies (12–48 h, 4 °C) against collagen 4 (COLL4; 1:5000, rabbit, Abcam, Cambridge, UK), CD68 (1:2000, mouse, DAKO, Glostrup, Denmark) or glial fibrillary acidic protein (GFAP; 1:1000, rabbit, Abcam, Cambridge, UK) and a secondary biotinylated antibody (1:200; 2 h, room temperature, Vector Laboratories, Burlingame, CA, USA). Alternatively, sections were incubated for 48 h with *Ulex europaeus* lectin (UEA-l; 1: 800, biotin-coupled, GeneTex, Irvine, CA, USA). Immunohistochemical reactions or lectin binding were visualized with an avidin-biotin-peroxidase complex (ABC Vectastain, Vector Laboratories, Burlingame, CA, USA) and 3,3′-diaminobenzidine tetrahydrochloride (DAB; Sigma Taufkirchen, Germany). For double-label immunohistochemistry, sections were washed with TBS at 95 °C for 5 min, and the procedure was repeated using the next primary and secondary antibody. Subsequently, a blue chromogen (Vector SK-4700 peroxidase substrate kit, Linaris, Doffenheim; Germany) was used to visualize the reaction product. Omission of the lectin or primary antibody resulted in lack of staining.

The Coll4-positive microvasculature was evaluated in all microinfarcts, which had been identified in neighboring PN and H&E stained sections (see Table 1). In addition, histological characteristics of microinfarcts were studied using double-label-IHC for Coll4 and GFAP (9 sections, in 6 cases), for Coll4 and CD68 (14 sections, in 6 cases), for Coll4 and Aβ (8 sections, in 7 cases) and for Coll4 and UEA-l (8 sections, 4 cases). MRI-scanned sections were stained using Coll4-/UEA-double-labeling (*n* = 5), H&E (*n* = 2), Prussian blue (*n* = 1), and Coll4-/CD68-double-label-IHC (*n* = 2).

### Image acquisition and processing

Dicom images were viewed with the software RadiAnt DICOM Viewer (version 4.6.5) and Image J (version v1.52j) and exported in tiff-format. Brightness and contrast of images were optimized with the GNU Image Manipulation Program (GIMP - version 2.8.16) and Adobe Photoshop (version 10.0). The tissue object was segmented and presented on a black background for an optimal comparability of the different scan sequence contrasts. To visualize the microinfarct zone in selected areas and insets, images were re-scaled by cubic interpolation. Enlarged images were smoothened using Gaussian blurring and/or despeckling. Microphotographs of histological sections were taken with a digital camera (Jenoptik Progres Gryphax® Prokyon, Jena, Thüringen, Germany) using an AX10 microscope (Zeiss, Jena, Germany). For the detection of lipofuscin autofluorescence, sections were imaged with the aid of a LED fluorescence lamp and narrow selective green H bandpass filter set (F46–801, AHF Analysetechnik, Tübingen, Germany). In IHC-stained sections, either single images were taken or z-stacks were obtained with bright field microscopy. Images were processed with the software Adobe Photoshop (version 10.0) to optimize light and contrast conditions as well as color temperature, hue and saturation. Multiple single images and z-stacks were stitched to visualize larger areas or merged in the z-dimension as needed.

## Results

### Chronic cortical cerebral microinfarcts contained extracellular pigment granules

**Fig. 2:**
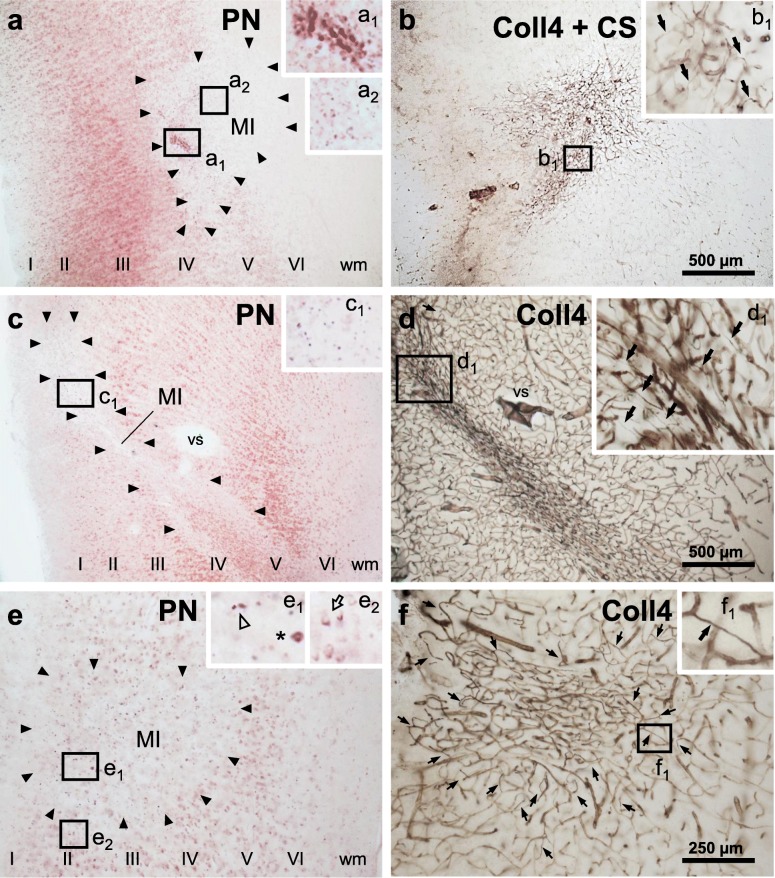
Localization of *chronic* microinfarcts in different cortical layers with cell loss in the pigment Nissl (PN) stain and microvascular changes shown with collagen 4 (Coll4)-immunohistochemistry in two adjacent thick brain sections. **a**, **c**, **e** Microinfarcts are located at deep cortical layers, often extending to the juxtacortical white matter (**a-b**; superior frontal microinfarct, case 6), at superficial cortical layers (**e-f**; anterolateral superior frontal microinfarct, case 6) or transcortically (**c-d**; parietooccipital microinfarct, case 4). The core of the microinfarction zone (MI) exhibits pallor in the PN stain due loss of Darrow red-stained neurons (boundaries of the MI indicated by black arrow heads). The MI core further contains aldehyde fuchsine-stained microglial cells (inset **a**_**1**_; asterisk in inset **e**_**1**_; also see Braak et al., 2018) and, as a result of cell loss, extracellular lipofuscin granules (insets **a**_**2**_ and **c**_**1**_; open arrow head in inset **e**_**1**_) that are easily distinguishable from intracellular lipofuscin granules seen in surviving Darrow red-positive neurons outside the MI zone (open arrow in inset **e**_**2**_). **b**, **d**, **f** In the MI core, *chronic* microinfarcts display a dense Coll4-immunoreactive microvascular meshwork (**b** double-labeled for Campbell-Switzer, CS) with numerous string vessels (black arrows in insets **b**_**1**_ and **d**_**1**_). String vessels also decorate the zone surrounding the MI core (black arrows in **f**; black arrow in inset **f**_**1**_). Scale bars: 1000 μm (**a-d**) and 2000 μm (**e-f**)

**Fig. 3:**
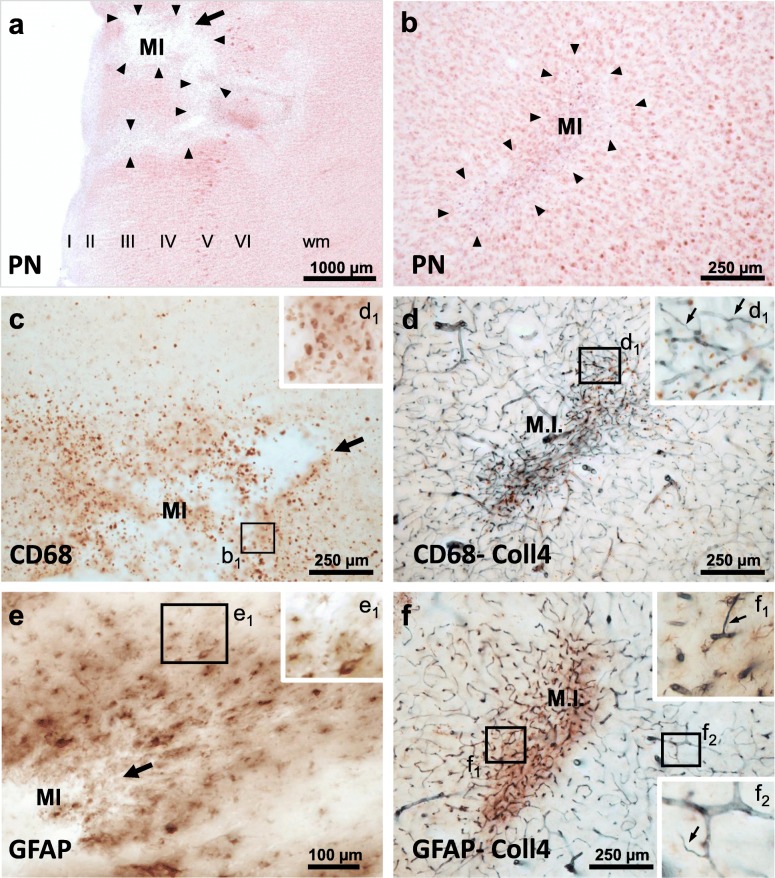
Chronic cortical cerebral microinfarct invaded by CD68-expressing macrophages with a phagocytic phenotype and glial fibrillary acid (GFAP)-expressing astrocytes. Example of cavitated cortical cerebral microinfarct with puckering that is located superficially (**a**, **c** and **e**; superior frontal, M1 motor cortex) and a second microinfarct occupying mid cortical layers (**a**, **c** and **e**; medial side of middle frontal gyrus) from the same case (case 1). **a-b** The PN stain shows cell loss in both microinfarcts (**a**, **b**) and cavitation in the superficial microinfarct that partially extends to deeper cortical layers (**a**). **c-d** Relationship of CD68--immunoreactive macrophages and changes in the microvasculature in the core region of the MI (**c**, **d**). **e-f** GFAP-positive astroglial cells infiltrate both the core and marginal zone of the MI and are not restricted to the zone with the dense Coll4-immunoreactive microvascular meshwork. Moreover, both the core and periphery of the MI are covered with numerous string vessels (insets **d**_**1**_ and **f**_**1–2**_). Scale bars: 2000 μm (**a**), 800 μm (**b-d**, **f**) and 400 μm (**e**)

**Fig. 4:**
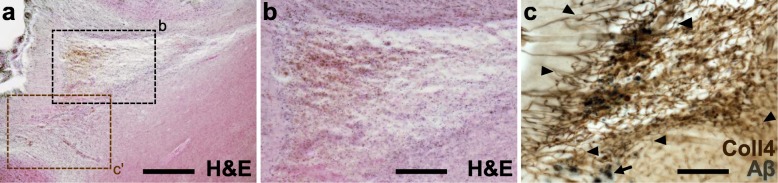
Modified H&E stain (case 5) showing a *chronic* cortical cerebral microinfarct with comparatively large tissue thinning and cavitation in the occipital lobe, which extends from mid-cortical layers to the juxtacortical zone (**a**-**b**). In a neighboring section double-labeled for Coll4 and beta-amyloid (Aβ), the same microinfarct displayed a dense microvascular meshwork with numerous string vessels (arrow heads) and parenchymal Aβ deposits (arrow) (**c**). The inset (c’) in the H&E-stained section (h) corresponds approximately to the same area as in (**c**). Scale bars: 500 μm (**a**) and 200 μm (**b**-**c**)

**Fig. 5:**
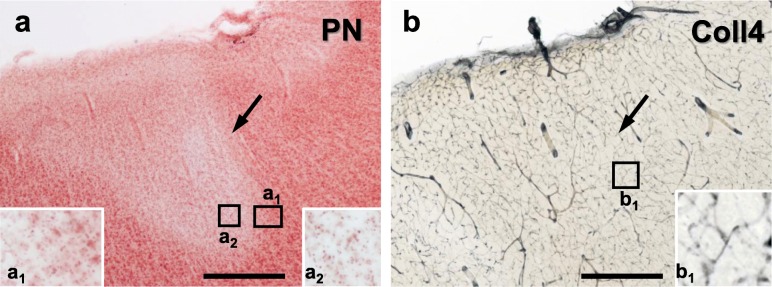
Subacute superficial cortical cerebral microinfarct (case 5) with pallor in the PN stain (**a**). At higher magnification, neuronal somata show reduced Darrow red (Nissl) staining, but no extracellular lipofuscin granules are visible (**a**_**1**_ and **a**_**2**_). Also, no string vessels or other obvious changes are evident in the microvascular network visualized with IHC using antibodies against Coll4 (**b** and **inset b**_**1**_). Scale bars: 500 μm (**a-b**)

Cortical cerebral microinfarcts were found in cases with and without cortical Aβ deposits, hence, also in the absence of cerebral amyloid angiopathy (CAA) (Table 1). Microinfarcts could be easily detected by screening the cerebral cortex in PN-stained sections [8]. The PN stain also helped to distinguish (sub)acute microinfarcts from microinfarcts at chronic stages. The latter microinfarcts, which were now called *chronic cortical cerebral microinfarcts* here, stood out in the PN stain through tissue pallor and lipofuscin pigment accumulation (Fig. 2a, c and e; Fig. 3a-b). In all *chronic* microinfarcts studied, lipofuscin granules formed disorganized aggregates outside the cytoplasm of Darrow red-stained neurons or other cells (Fig. 2c1 and e1). The disorganized lipofuscin granules were located in areas with neuronal cell loss showing pallor. Hence, they resembled extraneuronal lipofuscin remnants typically found in areas with extended neuronal cell loss in other neurological diseases (e.g., [5, 8, 16, 54]). The extracellular lipofuscin aggregates in microinfarcts could be clearly distinguished from intraneuronal lipofuscin granules (Fig. 2e2, see also [44]) or the purplish, cytoplasmic aldehyde fuchsine staining seen in macrophages that clustered in the microinfarct core (Fig. 2a1, see also [8]). In the H&E stain adapted to thick sections, *chronic* microinfarcts also displayed pallor and tissue thinning (Fig. 4 and Supplementary file S1). However, pallor was mild compared to the PN stain in small cortical infarcts with minor tissue thinning, making it difficult to detect the microinfarction zone (Supplementary file S1). (Sub)acute ischemic cortical cerebral microinfarcts, by contrast, only exhibited pallor of the Nissl substance in the absence of extracellular lipofuscin accumulation (Fig. 5a, case 5, died shortly after admission to hospital).

### Chronic microinfarcts contained a dense microvascular meshwork with string vessels

**Fig. 6:**
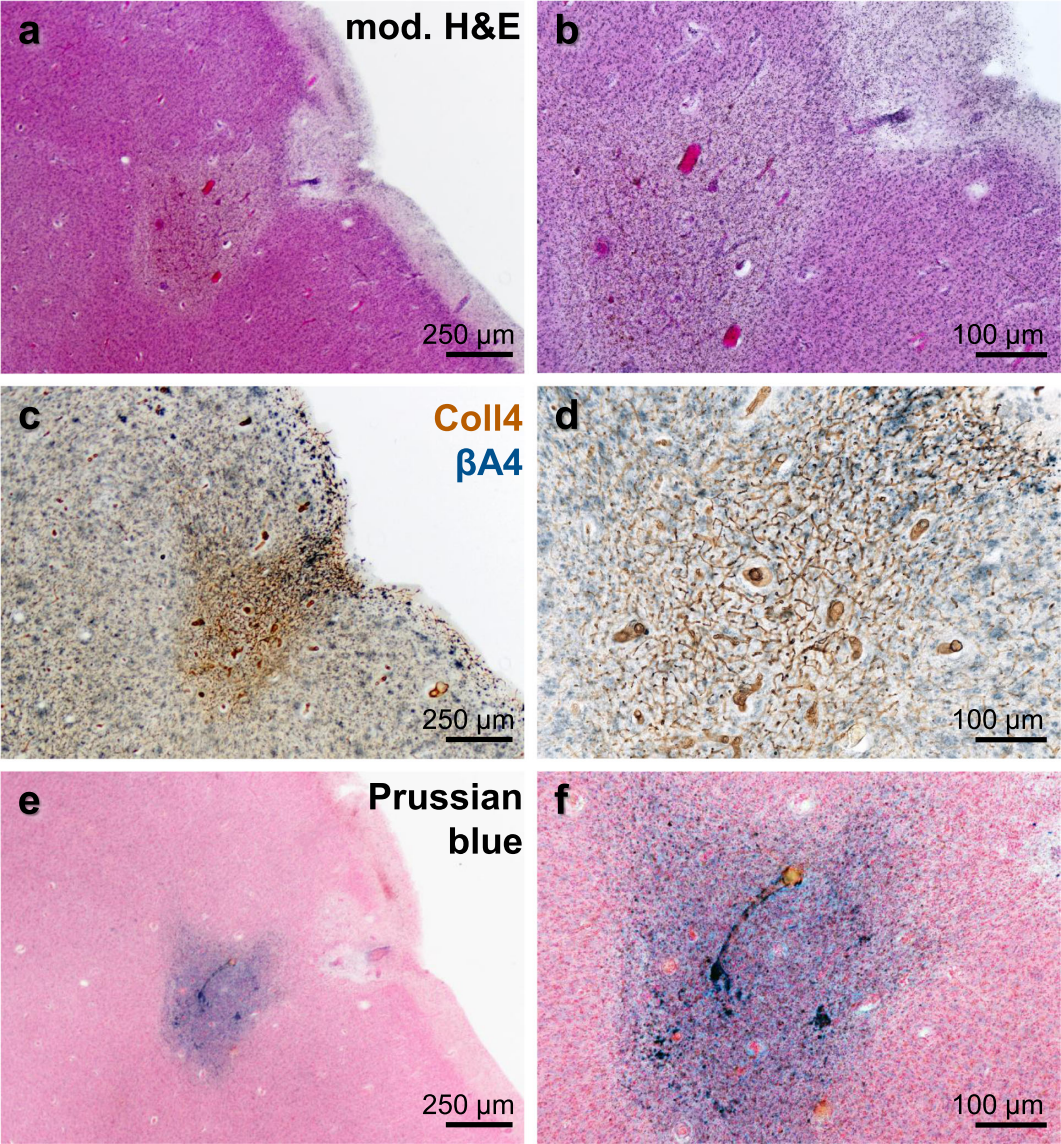
Morphological characteristics of a superficial cortical microinfarct shown in three adjacent thick sections from a case with diffuse parenchymal Aβ deposits (case 8). **a-b** The microinfarction zone shows pallor and altered microvessels in the modified H&E stain. **c-d** Microvascular changes in this case consist of a dense microvascular meshwork with a high density of string vessel as seen in cases without Aβ deposits. **e-f** Prussian blue staining shows iron accumulation in the core of the microinfarction zone. Scale bars: 250 μm (**a**, **c** and **e**) and 100 μm (**b**, **d** and **f**)

A dense meshwork of Coll4-positive microvessels was found in all microinfarcts that fulfilled the criteria of *chronic* microinfarcts in the PN stain as described above. The dense microvascular meshwork covered the heart of the microinfarction zone with tissue pallor, which contained extracellular lipofuscin aggregates (Fig. 2a_2_, c_1_ and e_1_) and/or macrophages (Fig. 2a_1_ and e_1_; see also [8]). The dense microvascular meshwork also occupied the “cavitated” zone of *chronic* microinfarcts with puckering and cavitation (Figs. 4c and 10e). *Chronic* microinfarcts further exhibited many Coll4-positive string vessels, which correspond to thin connective tissue strands that are formed by collapsed basement scaffolds connected to the capillary network [10, 22]. Especially, the microinfarction zone covered with the dense microvascular meshwork contained numerous Coll4-positive string vessels. Moreover, string vessels were observed in the marginal zone of microinfarcts, which surrounded the area with the dense microvascular meshwork (Figs. 2 and 3). In double-labeled sections, Coll4-positive string vessels often lacked staining with the lectin UEA-l (see Fig. 10), which indicated endothelial cell damage, since UEA-l binds to the glycocalyx on the luminal surface of vascular endothelial cells [22]. The dense microvascular meshwork with string vessels was also found in a cortical microinfarct with heavy iron accumulation, although this case exhibited diffuse cortical Aβ deposits and no CAA (Fig. 6c-f). Such microvascular changes were absent in (sub)acute ischemic cortical cerebral microinfarcts (Fig. 5).

### Chronic microinfarcts are infiltrated by CD68- and GFAP-positive cells

In all *chronic* cortical cerebral microinfarcts studied, the core of the infarction zone was invaded by numerous CD68-positive macrophages, which coincided with the area covered by the dense microvascular meshwork (Fig. 3c-d). The infarction zone of all *chronic* microinfarcts also contained a prominent astrocytic scar (Fig. 3e-f). Both cell types were not limited to the area with the highest microvascular density. They also covered the margin of microinfarcts and some cells extended to the neuropil surrounding the microinfarct. Moreover, astrocytes surrounding microinfarcts with cavitation appeared larger than astrocytes in microinfarcts with minor thinning of the tissue (Fig. 3e).

### Cortical cerebral microinfarctions occur in different cortical layers

**Fig. 7:**
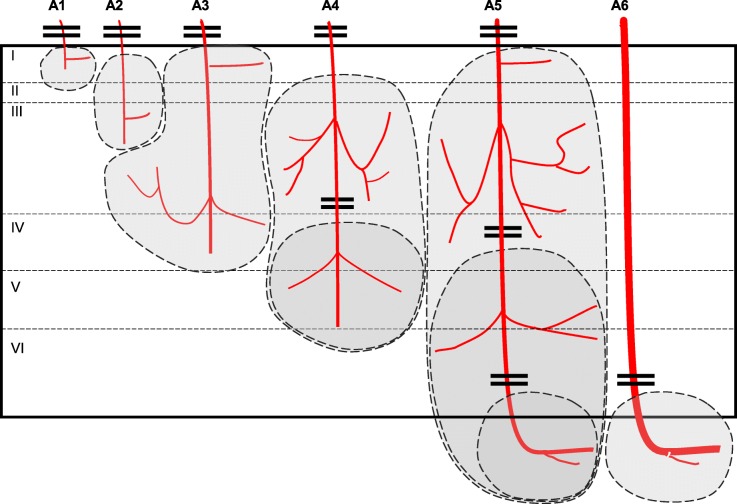
Diagram showing the blood supply of the cerebral cortex by A1 – A6 type arterioles according to Duvernoy et al. [17] and putative microinfarction zones resulting from the occlusion of these arterioles. According to this diagram, occlusion of A1 – A3 arterioles leads to superficial cortical microinfarcts, whereas the closure of deeper portions of type A4 – A6 arterioles give rise to deep cortical microinfarcts. When A4 – A5 arterioles are affected more superficially, this may cause a columnar microinfarct encompassing both superficial and deep cortical layers. Microinfarcts at juxtacortical positions may result from occlusion of deep branches of A5 – A6 arterioles, which supply the transition zone between the deep cortical layer and the underlying subcortical white matter

The size and location of the microinfarcts was determined using corresponding PN-stained and Coll4-labeled sections. The area that showed overlap of tissue pallor in the PN stain (Fig. 2a, c and e) and a dense Coll4-positive microvascular meshwork was defined as the microinfarction zone (Fig. 2b, d and f). Microinfarcts ranged from 200 μm to 2000 μm in width and/or length and were identified in all layers of the cerebral cortex (Supplementary file S2). Superficial and deep cortical microinfarcts were often broader, whereas transcortical microinfarcts were usually narrow. Superficial cortical microinfarcts frequently led to an indentation of the cortical surface. Deep infarctions with a juxtacortical position extended to the adjacent white matter (Fig. 2). Microinfarct locations were compatible with the layer-specific blood supply of the cerebral cortex by arterioles classified as A1 to A6 type arterioles by Duvernoy and colleagues [17]. Moreover, some transcortical microinfarctions bifurcated in deeper cortical layers indicating that both the main arteriolar branch and its side branches were affected (Fig. 2c). Thus, a disturbance in the circulation in A1–4 arterioles (e.g., through hypoperfusion, occlusion or thromboembolic closure) might lead to microinfarcts in superficial and middle cortical layers, whereas a cessation of the circulation in A4–6 arterioles could lead to microinfarcts in deep cortical and juxtacortical layers, and a full disruption of A5 arterioles to transcortical microinfarcts (Fig. 7).

### Variability of single section ultra-high resolution MRI signal intensities among microinfarcts

**Fig. 8:**
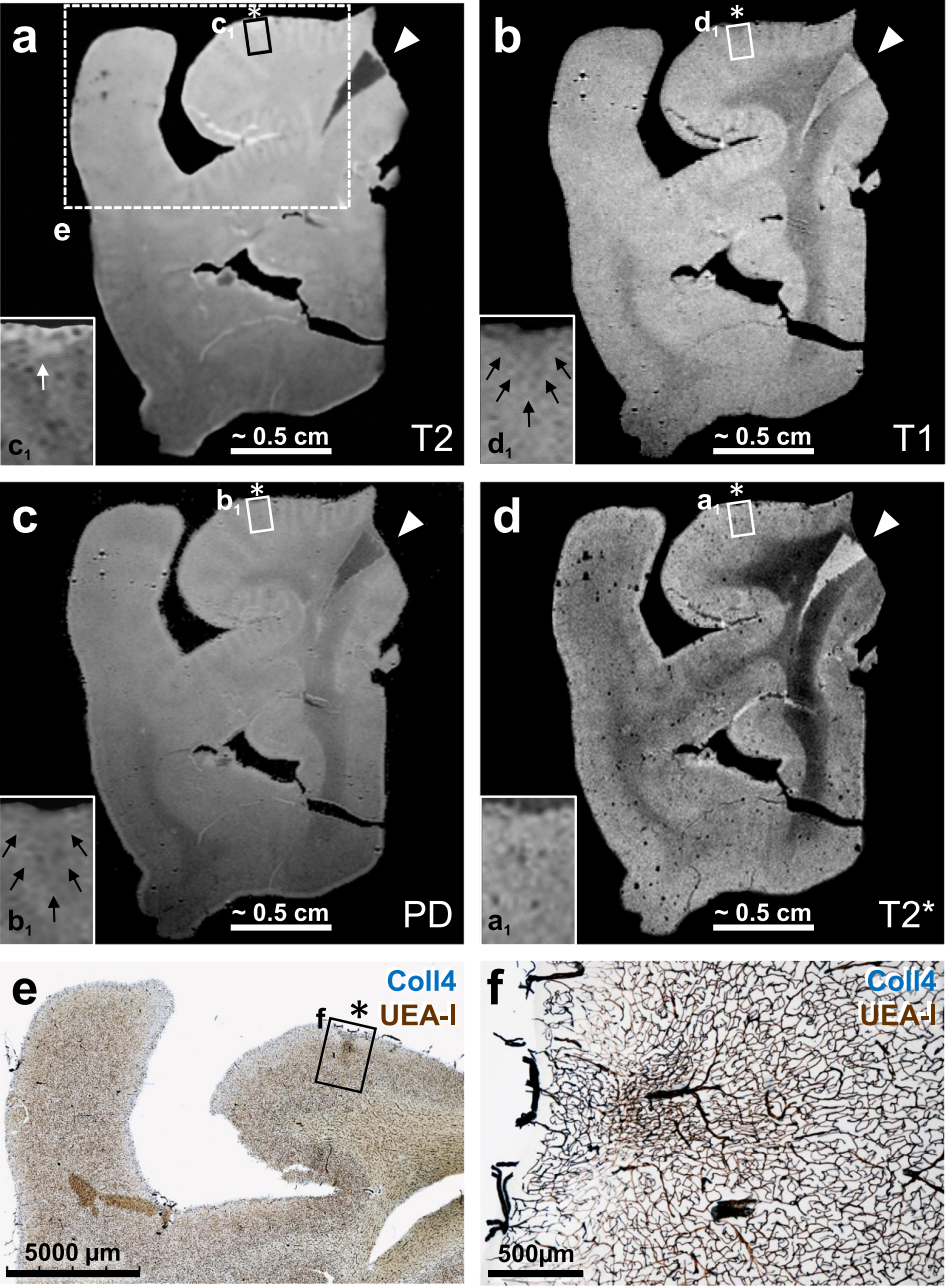
Postmortem ultra-high-resolution MRI of a 100 μm thick section scanned at 11.7 T followed by post-imaging histological staining (case 2). **a** The microinfarct (asterisk) could not be identified in the T2*-weighted image, and the microinfarction zone showed very little iron deposition as indicated by Prussian blue staining in an adjacent section (not shown). However, microvessels appeared enlarged due to the blooming effect, probably induced by susceptibility effects of iron magnetism in erythrocytes (see inset **a**_**1**_). **b** Proton density (PD) image sequence reflecting the proton (water) content in the tissue also failed to visualize this microinfarct (see asterisk and inset **b**_**1**_). **c** In the T2-weighted image, a slightly enhanced hyperintense signal was observed at the superficial part of the infarction zone (white arrow in inset **c**_**1**_). **d** The superficial zone of the microinfarct (see asterisk) also gave a slightly hypointense signal in the T1-weighted image (area indicated by black arrows in inset **d**_**1**_). **e-f** The MRI-scanned section showed a microinfarct with characteristic changes in the microvasculature as seen in double-labeling for Coll4 and UEA-l lectin, which visualizes the endothelial glycocalyx, but the microinfarct area covered by the dense microvascular meshwork could not be distinguished well from the surrounding tissue based on MRI signals [see in frame **f** in (**e**) and the enlarged inset in (**f**)]. Arrow heads (**a**-**d**) points to an artefact resulting from an overlay of two flee-floating ends of the section. Scale bars: 3 mm (**e**) and 500 μm (**f**)

**Fig. 9:**
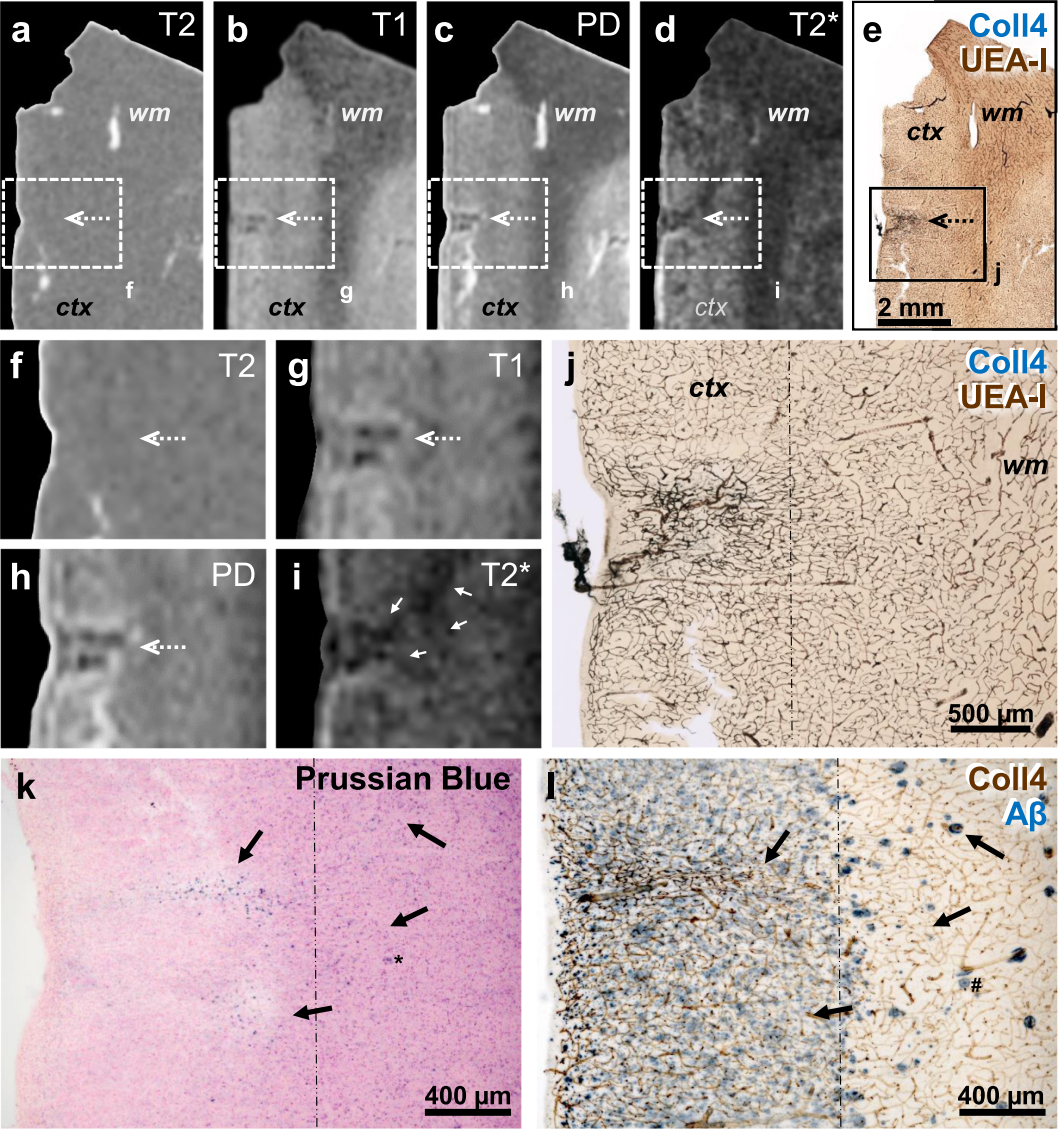
Thick brain section (case 8) first scanned with 11.7 T MRI and then double-labeled for Coll4 and UEA-l (**a-j**), whereas adjacent sections were stained with iron histochemistry (**k**; Prussian blue stain) and double-label immunohistochemistry for Coll4 and Aβ (**l**). **a**, **f** T2-weighted image does not show changes in the signal intensity in the microinfarct area (dotted arrow) despite the strong hyperintense signal produced by saline in a tear nearby and around the section. **b**, **g** The same microinfarct (dotted arrow) gave a hypointense T1-weighted signal. **c**, **h** In the PD-weighted image, the microinfarct gave a reduced and its margin a hyperintense proton signal. **d-e** and **i-j** The hypointense microinfarct zone with a blooming effect in the T2*-weighted image (**d**, **i**) contained a dense microvascular meshwork (**e**, **j**). **k-l** Microinfarct area giving a hypointense T2* signal (white arrows) showed iron deposition in the adjacent section (black arrows) independently from the diffuse Aβ deposition in superficial cortical layers, although larger iron aggregates in deep cortical layers (asterisk in **k**) may be localized in cored Aβ plaques (hashtag in **l**). *ctx* cortex; *wm* white matter. Scale bars: 2 mm (**e**), 500 μm (**j**) and 400 μm (**k-l**)

**Fig. 10:**
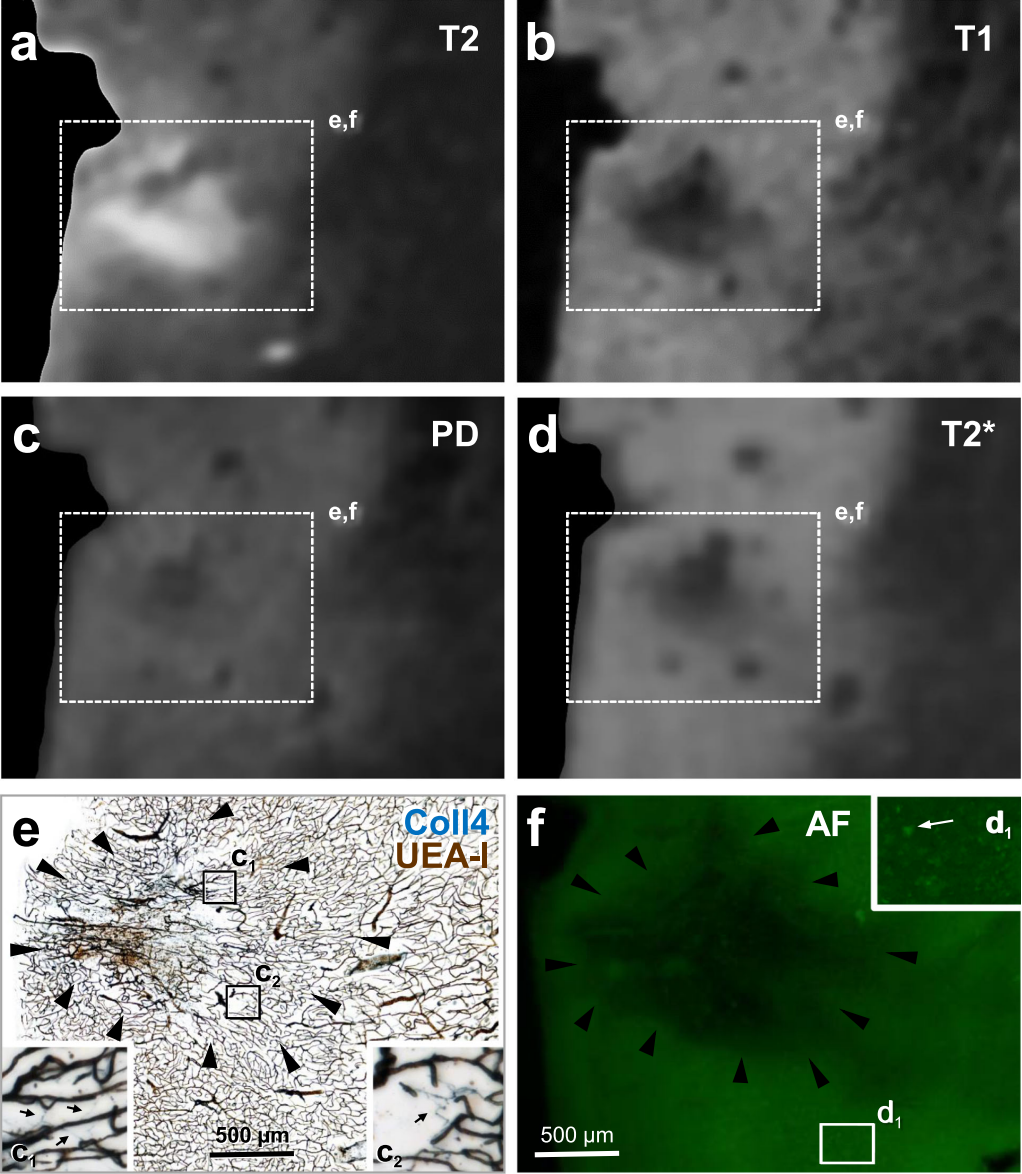
Images from a 100 μm-thick brain section with a superficial cavitated microinfarct in the motor cortex (case 1). The section was first scanned with 11.7 T MRI (**a-d**) and then double-labeled for Coll4 and UEA-l (**e**) after capturing autofluorescence (**f**). **a** The cavity of the microinfarct gave a hyperintense signal in the T2-weighted image. **b** The T1-weighted MRI signal was hypointense in the microinfarct cavity and in tissue areas that showed thinning. **c** The PD-weighted image exhibited a hypointense signal in the core and a slightly hyperintense signal in the rim of the microinfarct cavity. **d** In the T2*-weighted image, large vessels and the microbleed in the cavity produced magnified hypointense signals (blooming effect). **e** The microinfarct cavity contained damaged microvessels and the rim of the cavity was rich in Coll4-positive string vessels that lacked UEA-l staining. **f** Autofluorescence (AF) of the tissue was reduced in the microinfarct cavity and in areas with tissue thinning around the cavity. Betz cells could also be identified based on their autofluorescence (arrow in inset **d**_**1**_). Scale bars: 500 μm (**e-f**)

Altogether 6 histologically verified microinfarcts were identified in 5 MRI-scanned sections (Table 1). The microinfarct area, which showed pallor in the PN stain and was covered by a dense microvascular meshwork in the histological sections, produced hypointense T1- and PD-weighted MRI signals and hyperintense T2-weighted MRI signals. Moreover, in T2*-weighted gradient echo images, a hypointense signal with a blooming effect was seen in microinfarct zone, which probably originated from paramagnetic signals produced by iron aggregates. However, the intensity of MRI signals produced by the *chronic* cortical cerebral microinfarcts varied considerably, although in histological sections all microinfarcts exhibited similar pathological features, such as a dense Coll4-positive microvascular meshwork with string vessels, loss of Nissl-stained neurons, and extracellular lipofuscin granules. The intensity of T1-weighted and PD-weighted MRI signals of microinfarcts ranged from low (Fig. 8; Supplementary files S3 and S4) to strong (Figs. 9 and 10). A stronger T1- or T2-weighted hypo-/hyperintense signal correlated with tissue thinning and cavitation (Fig. 10). In some microinfarcts, the rim of the microinfarction zone gave a slightly hyperintense MRI signal in PD-weighted images (Fig. 10c, Supplementary file S3). Furthermore, the intensity of T2*-weighted MRI signals correlated more with the level of iron accumulation in the microinfarct core rather than the density of parenchymal diffuse or cored Aβ plaques. In T2*-weighted images, microinfarcts with a low iron load produced a low signal contrast (Fig. 8, Supplementary file S3). In microinfarcts with iron accumulation, the intensity of the hypointense T2*-weighted MRI signal was higher in microinfarcts with a higher iron load when compared to microinfarcts with low iron accumulation, as can be seen in two different microinfarcts from the same case (Fig. 9 and Supplementary file S4).

## Discussion

The microvasculature at the infarction zone of *chronic* cortical cerebral microinfarcts showed unique pathologic features that seemed to be largely independent of their location in different layers of the cerebral cortex. Common to all *chronic* microinfarcts identified in this study was tissue pallor with loss of neurons and lipofuscin pigment accumulation that was accompanied by a dense microvascular meshwork. The microvascular meshwork in the microinfarct core and the microinfarct margin contained a high density of string vessels that lacked expression of the endothelial marker UEA-l. In addition, the microinfarct core was infiltrated by activated CD68-positive macrophages and GFAP-positive astrocytes, whereas astrocytes often extended to the microinfarct periphery. Nevertheless, *chronic* cortical cerebral microinfarcts also showed some variability, especially with regard to their size and shape and the degree of iron accumulation in the microinfarct core, which impacted their detectability in postmortem ultra-high-resolution MRI.

These features of *chronic* cortical microinfarcts are consistent with findings obtained in thin paraffin sections stained for IHC and/or H&E, which displayed pallor due to reduced eosinophilic staining of the neuropil and loss of neurons, astrogliosis, activated microglial cells, and occasionally cystic formations [12, 15, 26]. (Sub)acute cortical cerebral infarcts did not exhibit these pathological changes or a dense microvascular meshwork, thereby supporting the chronic nature of the microinfarcts imaged here [13, 33, 34]. Therefore, these microinfarcts were summarized as “*chronic*” microinfarcts now, although the observed pathologies may represent different phases of microinfarcts. In patients with stroke, where the sequence of pathological events in infarcts could be established based on the timepoint of injury, three phases were described: (1) Acute neuronal injury with cytoplasmic vacuolation, eosinophilia (red neurons), and ghost cell formation together with edematous spongiosis, (2) necrosis and organization, which was characterized initially by (a) acute inflammation with neutrophils, and later by (b) chronic inflammation with macrophages and perivascular cuffing, and finally (3) chronic resorption with macrophages, pallor, spongiosis and/or cavitation. Phases 2 and 3 also exhibited various degrees of axonal spheroids, gemistocytes, and neovascularization [34].

Larger and/or cavitated cortical cerebral microinfarcts were detectable in histological sections in the PN stain, Coll4-IHC, iron histochemistry and H&E staining adapted to thick brain sections. However, smaller microinfarcts with minor tissue thinning in the absence of cavitation or puckering were often barely noticeable in thick H&E-stained sections. Therefore, thick brain sections stained for PN stain and Coll4-IHC were screened for a more reliable identification and characterization of the latter microinfarcts. Microinfarcts are common in patients with mild cognitive impairment and dementia, but current estimates of their numbers in the human brain are largely based on the extrapolation of findings obtained in standard paraffin sections [12, 48]. Therefore, the insights gained here into the organization of smaller cortical cerebral microinfarcts provide a basis for a more systematic investigation of cortical microinfarcts in larger patient cohorts. Our analyses of thick brain sections further allowed the reliable detection of comparatively small iron depositions in microinfarcts, although factors that influenced iron accumulation in *chronic* microinfarcts remained elusive. At chronic phases of microinfarcts, activated macrophages can persist in the core of the infarction zone, as shown here and by others [42, 50], where they may engulf extracellular lipofuscin aggregates resulting from neuronal cell death [8] or take up iron in response to neuroinflammatory processes [39]. Moreover, the microinfarct zone containing iron accumulation coincided with the zone infiltrated by activated microglial cells and a dense microvascular meshwork. Inasmuch as these cortical microinfarcts might have originally evolved from ischemic [2, 32] or hemorrhagic infarcts, a major source of iron in microbleeds is hemoglobin and its degradation products, which lead to transformation of macrophages into hemosiderophages [18, 31].

On postmortem MR images obtained with a field strength of 1 Tesla (T), only 25% of cortical microinfarcts were detected correctly and 25% were thought to be artifacts, whereas 33% of the histologically verified microinfarcts were not visible [19]. The detection rate of cortical microinfarcts was also improved considerably by imaging at 7 T in comparison to 3 T, which was confirmed histologically through subsequent serial sectioning. Therefore, technical limitations, such as field strength, image resolution, pulse-sequence parameters and MRI-histological matching were considered to be the major sources for obtaining false negative results [15, 25, 47, 49]. However, our analyses with a field strength of 11.7 T now showed that there is also some variability in the histopathological features of cortical cerebral microinfarcts, including not only their size and shape, the cortical layer in which they occur, thinning, and cavitation, but also the degree of iron accumulation, which influenced MRI signals and the detectability of microinfarcts in this study. While microinfarcts produced hypointense MRI signals in T1-weighted, and to some extend also in PD-weighted images, hyperintense T2 signals were more variable. Moreover, the T1 signal strength was more prominent when thinning of tissue or cavitation was evident, and strong T2 signals were also observed in microinfarcts with cavitation. These MRI signal intensities and contrasts produced by cavitated and non-cavitated cortical cerebral microinfarcts at 11.7 T in T1-, T2, PD- and T2*-weighted sequences were consistent with data obtained at 7 T in previous MRI-histological correlation studies [15, 46, 49]. However, MRI signals of smaller and narrower microinfarcts with less tissue thinning could be also imaged and histologically studied here with 11.7 T MRI, although the MRI signals produced by these microinfarcts with lower field strengths or in vivo MRI scans remains to be determined. Iron accumulation, histologically confirmed with Prussian blue staining, was the likely source of changes in susceptibility detected in *chronic* microinfarcts in our T2*-weighted MR images. In clinical studies, T2*-weighted MRI sequences are commonly used to detect microbleeds in patients with CAA [53]. Of note, several cases here suffered from cerebrovascular diseases resulting in stroke and most of the individuals either lacked extracellular Aβ aggregates or they displayed mainly parenchymal Aβ deposits.

A major obstacle in analyses of structural correlates of MRI signals is the identification of the spatial correspondence of a lesion between the MR images and histological sections, commonly referred to as MRI-histological matching [1, 37]. Moreover, histological sections often show deformations that occur during resection, handling and histological processing of the tissue, making the registration of tissue-derived histological images to the MR images necessary. During registration, deformations in histological images are removed with algorithms used for remodeling and transformation of the images with the goal to improve the spatial correspondence between MRI scans and histological images [24, 37]. By imaging free-floating thick brain sections with a field strength of 11.7 T, we could not only obtain MR images with ultra-high resolution here, but also circumvent problems associated with MRI-histological matching and image registration. Furthermore, we could achieve a direct correlation between the MRI signals acquired and histological changes in the microinfarction zone including smaller iron deposits and string vessels that are difficult to detect in standard paraffin sections. Nevertheless, ultra-high-resolution imaging of 100 μm thick brain sections might result in a reduced MRI signal-to-noise ratio despite the use of high field strengths. Postmortem formalin fixation also alters MRI signals [36], potentially affecting the detection of structural changes in the density of microvessels, macrophages or astrocytes, especially in T1- and T2-weighted and PD images.

The blood supply of the cerebral cortex is provided by branches of major cerebral arteries that are located in the subarachnoid space. Arterioles arising from these branches enter gyri and sulci in a perpendicular fashion and give rise to branches that supply specific layers of the cerebral cortex. Duvernoy et al. [17] have classified cortical arterioles from A1-A6 based on the depth of the cortical layer they reach, which correlates well with the locations of the microinfarcts observed here. Thus, hypoperfusion or bleeding in the zones supplied by A1-A5 type arterioles or their branches is perfectly suited to cause circumscribed infarctions in the superficial and deep layers of the cerebral cortex. Because A5 type arterioles run through the cortex and reach the subcortical white matter, their occlusion may result in transcortical microinfarctions. Moreover, portions of A5 and A6 arterioles that reach the subcortical white matter form multiple anastomoses (Duvernoy at al., 1981). This may explain the confinement of microinfarcts to deep cortical and juxtacortical locations, and the sparing of the deeper subcortical white matter, when juxtacortical arteriolar branches are affected. Rupture or occlusion of these arterioles could be caused by CAA or atherosclerosis of leptomeningeal vessels and their arteriolar branches that supply the cerebral cortex [31, 43]. Other causes of cortical cerebral microinfarcts include thrombotic microembolisms originating from the heart (e.g., endocarditis) or plaques in cerebral arteries, lipid microembolisms following major surgeries, hereditary forms of small vessel disease such as CADASIL, leukoencephalopathy, and radiation therapy [11, 25].

A common feature of the *chronic* cortical cerebral microinfarcts seen here was the presence of a dense microvascular meshwork with string vessels in the infarction zone. String vessels are known to emerge during the course of vascular regression, which is tightly regulated during angiogenesis and neovascularization [30, 38, 51]. Development of string vessels can be also mediated by a loss of factors that promote endothelial cell survival and angiogenesis such as vascular endothelial growth factor (VEGF) [3, 10, 40]. In our cases, Coll4-positive string vessels at microinfarcts and their periphery lacked UEA-l expression indicating endothelial recession, which is associated with cessation of blood perfusion in the vessels affected [10, 22, 55]. Thus, the presence of string vessels pointed to disturbances in the microcirculation in and around cortical cerebral microinfarcts, which may explain the comparatively strong MRI signals obtained from relatively small cortical microinfarcts in vivo [27, 28, 32]. Thus, advanced techniques that detect changes in cerebral perfusion [20] and/or axonal disorganization [14] may further enhance the visualization of *chronic* cortical cerebral microinfarcts with in vivo MRI, especially when combined with techniques that use local perturbations of the MR phase to enhance T2* contrast, e.g., 3D T2*-weighted imaging with postprocessing susceptibility-weighted imaging [25], to distinguish microbleeds from calcifications.

## Conclusions

Our results indicate that *chronic* cortical cerebral microinfarctions occur at superficial, middle and deep layers of the cortex, and they can extend throughout all cortical layers, which is consistent with the pattern of blood supply provided by different types of cortical arterioles. Ultra-high resolution MRI performed in single sections permits the characterization of MRI signal contrast in small *chronic* cortical cerebral microinfarcts with minor thinning. Together with recent improvements in the resolution of imaging techniques in vivo, the knowledge of the location, shape and MRI signal contrast of cortical cerebral microinfarcts and patterns of iron accumulation may aid in the development of new analytical tools for their identification with imaging. Moreover, cortical cerebral microinfarcts at chronic stages exhibit a high vessel density with numerous string vessels in and near the microinfarct, which may alter the pattern of blood flow in the infarction zone. Such changes in the microcirculation in the microinfarction zone might be detectable with advanced imaging techniques, making it possible to identify cortical cerebral microinfarcts in living patients.

## Supplementary information

**Additional file 1: Figure S1.** A chronic cortical cerebral microinfarct in the superior frontal gyrus with minor tissue thinning that is located in mid-cortical layers (case 6) is shown in two neighboring 100 μm-thick brain sections stained with the pigment Nissl (PN) (**a**) and modified H&E stains (**b-c**). In thick brain sections, the boundaries of the pale microinfarct are clearly evident in the PN stain, but the paleness of the microinfarction zone is more difficult to discern in the modified H&E stain. The inset (**c**) shows an enlaged area from (**b**) with various cell types and vessels (arrow) in the core of the microinfarct stained with H&E. Scale bars: 400 μm (**a-b**) and 150 μm (**c**).

**Additional file 2.**

**Additional file 3: Figure S3.** The insets of MR images from Fig. 8 are presented here again (**a-d**) together with an autofluorescence image (AF) taken from the same 100 μm-thick section after the MRI scan (**i**). The microinfarct was also visualized in neighboring sections using single-labeling for Coll4 (**e**), Prussian blue staining (**f**), double-labeling for Coll4 and beta amyloid (Aβ) (**g**) and the pigment Nissl (PN) stain (**h**).

**Additional file 4: Figure S4.** MRI scans and histology of a second microinfarct from the same imaged (**a-j**) and same adjacent sections (case 8) shown in Fig. 8 (**k, l**). Although this microinfarct also contains a dense Coll4- and UEA-l-positive microvascular meshwork (**e, j**) and cortical Aβ deposit (diffuse superficially, cored in deep layers; **l**), its signal in T1-, T2- and PD-weighted images is difficult to detect (**a-c and f-h**). However, the T2*-weighted hypointense signal produced by the microinfarction zone is comparatively weak (**d, i**), which correlates well with the lower degree of iron accumulation at the microinfarction area (**k**) compared to the other microinfarct. Scale bars: 2 mm (**e**) and 500 μm (**j-l**).

## Data Availability

All data generated or analyzed during this study are included in this published article.

## Abbreviations

3D: Three-dimensional
ABC: Avidin-biotin-peroxidase complex
AD: Alzheimer’s disease
BSA: Bovine serum albumin
CAA: Cerebral amyloid angiopathy
CDR: Clinical Dementia Rating
COLL4: Collagen 4
DAB: Diaminobenzidine tetrahydrochloride
GFAP: Glial fibrillary acidic protein
H&E: Hematoxylin eosin
ICH: Immunohistochemistry
MMSE: Mini Mental Status Examination
MR: Magnetic resonance
MRI: Magnetic resonance imaging
NFT: Neurofibrillary changes
NSA: Number of signal averages
PD: Proton density
PEG: Polyethylene glycol
PN: Pigment Nissl stain
TBS: Tris-buffered saline
TE: Echo time
TR: Repetition time
UEA-l: *Ulex europaeus* lectin
