# Supplementary material for: Histological correlates of postmortem ultra-high-resolution single-section MRI in cortical cerebral microinfarcts
Source: Acta Neuropathol Commun. 2020 Mar 13;8:33. doi: 10.1186/s40478-020-00900-1 (PMC7071593; doi:10.1186/s40478-020-00900-1)
Supplement: Supplementary file 1 — Additional file 1: Figure S1. A chronic cortical cerebral microinfarct in the superior frontal gyrus with minor tissue thinning that is located in mid-cortical layers (case 6) is shown in two neighboring 100 μm-thick brain sections stained with the pigment Nissl (PN) (a) and modified H&E stains (b-c). In thick brain sections, the boundaries of the pale microinfarct are clearly evident in the PN stain, but the paleness of the microinfarction zone is more difficult to discern in the modified H&E stain. The inset (c) shows an enlaged area from (b) with various cell types and vessels (arrow) in the core of the microinfarct stained with H&E. Scale bars: 400 μm (a-b) and 150 μm (c). [file 40478_2020_900_MOESM1_ESM.pptx]

## Slide 1
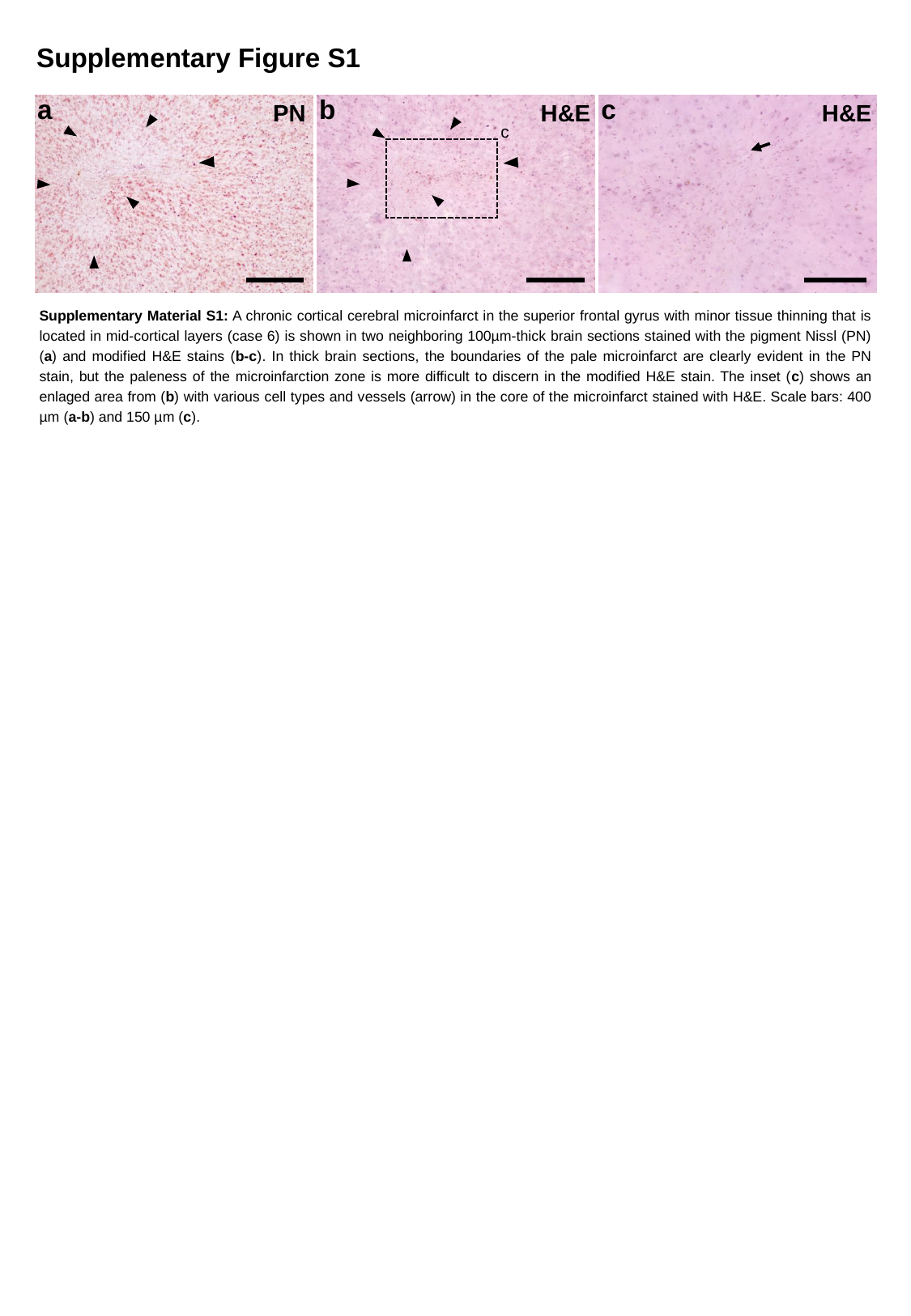

Supplementary Figure S1
a
b
c
PN
H&E
H&E
c
Supplementary Material S1: A chronic cortical cerebral microinfarct in the superior frontal gyrus with minor tissue thinning that is located in mid-cortical layers (case 6) is shown in two neighboring 100µm-thick brain sections stained with the pigment Nissl (PN) (a) and modified H&E stains (b-c). In thick brain sections, the boundaries of the pale microinfarct are clearly evident in the PN stain, but the paleness of the microinfarction zone is more difficult to discern in the modified H&E stain. The inset (c) shows an enlaged area from (b) with various cell types and vessels (arrow) in the core of the microinfarct stained with H&E. Scale bars: 400 µm (a-b) and 150 µm (c).
