# Supplementary material for: Histological correlates of postmortem ultra-high-resolution single-section MRI in cortical cerebral microinfarcts
Source: Acta Neuropathol Commun. 2020 Mar 13;8:33. doi: 10.1186/s40478-020-00900-1 (PMC7071593; doi:10.1186/s40478-020-00900-1)
Supplement: Supplementary file 3 — Additional file 3: Figure S3. The insets of MR images from Fig. 8 are presented here again (a-d) together with an autofluorescence image (AF) taken from the same 100 μm-thick section after the MRI scan (i). The microinfarct was also visualized in neighboring sections using single-labeling for Coll4 (e), Prussian blue staining (f), double-labeling for Coll4 and beta amyloid (Aβ) (g) and the pigment Nissl (PN) stain (h). [file 40478_2020_900_MOESM3_ESM.pptx]

## Slide 1
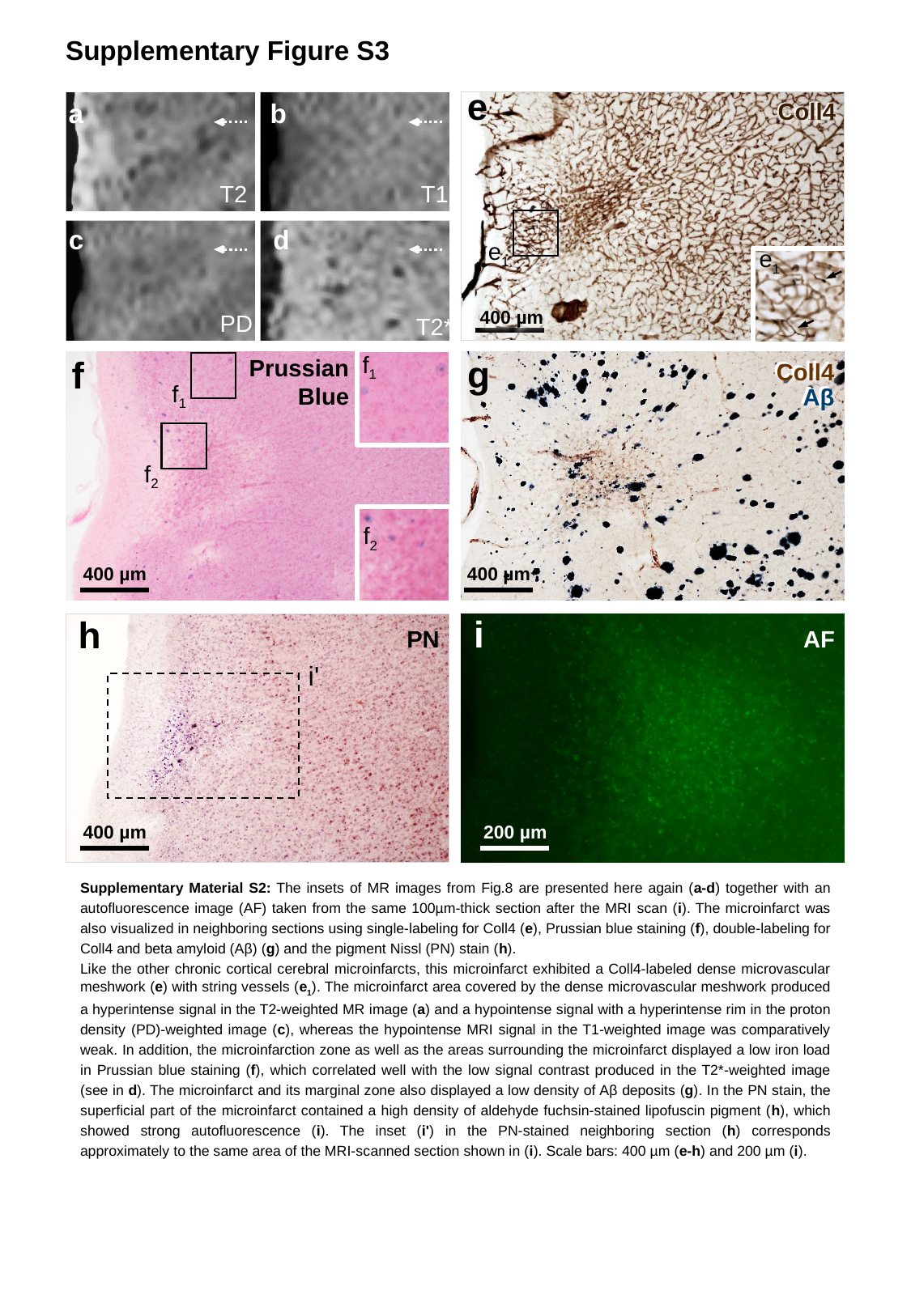

Supplementary Figure S3
e
Coll4
Coll4
a
b
T1
T2
c
d
e1
e1
400 µm
PD
T2*
f1
g
f
Prussian Blue
Coll4
Aβ
Coll4
Aβ
f1
f2
f2
400 µm
400 µm
i
h
PN
AF
i'
400 µm
200 µm
Supplementary Material S2: The insets of MR images from Fig.8 are presented here again (a-d) together with an autofluorescence image (AF) taken from the same 100µm-thick section after the MRI scan (i). The microinfarct was also visualized in neighboring sections using single-labeling for Coll4 (e), Prussian blue staining (f), double-labeling for Coll4 and beta amyloid (Aβ) (g) and the pigment Nissl (PN) stain (h).
Like the other chronic cortical cerebral microinfarcts, this microinfarct exhibited a Coll4-labeled dense microvascular meshwork (e) with string vessels (e1). The microinfarct area covered by the dense microvascular meshwork produced a hyperintense signal in the T2-weighted MR image (a) and a hypointense signal with a hyperintense rim in the proton density (PD)-weighted image (c), whereas the hypointense MRI signal in the T1-weighted image was comparatively weak. In addition, the microinfarction zone as well as the areas surrounding the microinfarct displayed a low iron load in Prussian blue staining (f), which correlated well with the low signal contrast produced in the T2*-weighted image (see in d). The microinfarct and its marginal zone also displayed a low density of Aβ deposits (g). In the PN stain, the superficial part of the microinfarct contained a high density of aldehyde fuchsin-stained lipofuscin pigment (h), which showed strong autofluorescence (i). The inset (i') in the PN-stained neighboring section (h) corresponds approximately to the same area of the MRI-scanned section shown in (i). Scale bars: 400 µm (e-h) and 200 µm (i).
