# Supplementary material for: Histological correlates of postmortem ultra-high-resolution single-section MRI in cortical cerebral microinfarcts
Source: Acta Neuropathol Commun. 2020 Mar 13;8:33. doi: 10.1186/s40478-020-00900-1 (PMC7071593; doi:10.1186/s40478-020-00900-1)
Supplement: Supplementary file 4 — Additional file 4: Figure S4. MRI scans and histology of a second microinfarct from the same imaged (a-j) and same adjacent sections (case 8) shown in Fig. 8 (k, l). Although this microinfarct also contains a dense Coll4- and UEA-l-positive microvascular meshwork (e, j) and cortical Aβ deposit (diffuse superficially, cored in deep layers; l), its signal in T1-, T2- and PD-weighted images is difficult to detect (a-c and f-h). However, the T2*-weighted hypointense signal produced by the microinfarction zone is comparatively weak (d, i), which correlates well with the lower degree of iron accumulation at the microinfarction area (k) compared to the other microinfarct. Scale bars: 2 mm (e) and 500 μm (j-l). [file 40478_2020_900_MOESM4_ESM.pptx]

## Slide 1
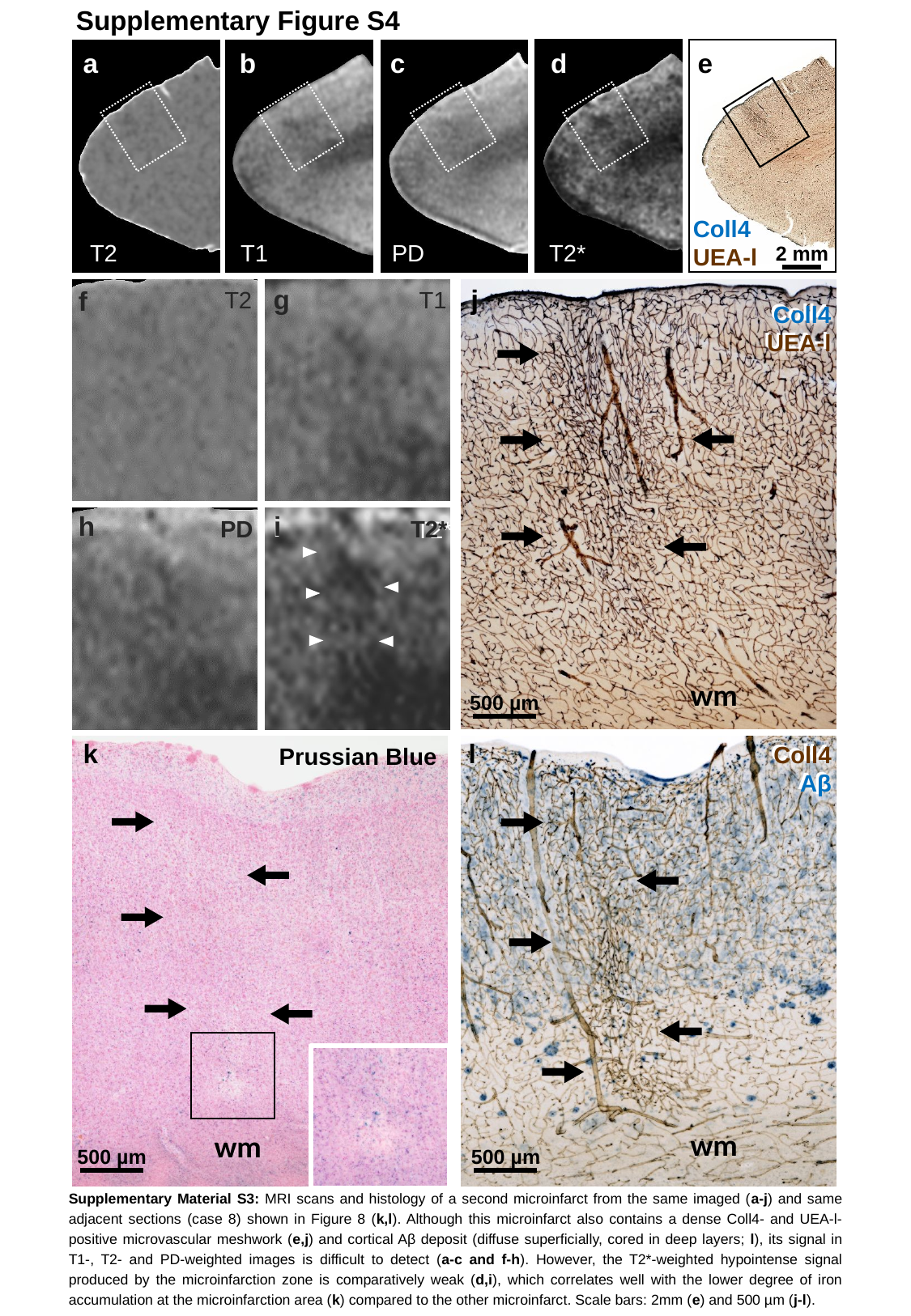

Supplementary Figure S4
a
b
c
d
e
Coll4
UEA-l
T2
T1
PD
T2*
2 mm
g
j
j
f
T1
T2
Coll4
UEA-l
Coll4
UEA-l
i
T2*
h
i
T2*
PD
wm
500 µm
k
l
Coll4
Aβ
Coll4
Aβ
Prussian Blue
wm
wm
500 µm
500 µm
Supplementary Material S3: MRI scans and histology of a second microinfarct from the same imaged (a-j) and same adjacent sections (case 8) shown in Figure 8 (k,l). Although this microinfarct also contains a dense Coll4- and UEA-l-positive microvascular meshwork (e,j) and cortical Aβ deposit (diffuse superficially, cored in deep layers; l), its signal in T1-, T2- and PD-weighted images is difficult to detect (a-c and f-h). However, the T2*-weighted hypointense signal produced by the microinfarction zone is comparatively weak (d,i), which correlates well with the lower degree of iron accumulation at the microinfarction area (k) compared to the other microinfarct. Scale bars: 2mm (e) and 500 µm (j-l).
